# Rural Financial Development Impacts on Agricultural Technology Innovation: Evidence from China

**DOI:** 10.3390/ijerph18031110

**Published:** 2021-01-27

**Authors:** Yuyu Liu, Duan Ji, Lin Zhang, Jingjing An, Wenyan Sun

**Affiliations:** 1 School of Accounting, Shandong University of Finance and Economics, Jinan 250014, China; 20188181@sdufe.edu.cn (Y.L.); 20174543@sdufe.edu.cn (D.J.); 2 School of Law, Shandong University of Technology, Zibo 255000, China; 3 Agricultural Bank of China, Qingdao 266000, China; sunwenyankr@abchina.com

**Keywords:** rural financial development, agricultural technology innovation, sustainable development in agriculture, public health

## Abstract

Agricultural technology innovation is key for improving productivity, sustainability, and resilience in food production and agriculture to contribute to public health. Using panel data of 31 provinces in China from 2003 to 2015, this study examines the impact of rural financial development on agricultural technology innovation from the perspective of rural financial scale and rural finance efficiency. Furthermore, it examines how the effects of rural financial development vary in regions with different levels of marketization and economic development. The empirical results show that the development of rural finance has a significant and positive effect on the level of agricultural technology innovation. Rural finance efficiency has a significantly positive effect on innovation in regions with a low degree of marketization, while the rural financial scale has a significantly positive effect on technological innovation in regions with a high degree of marketization. Further analysis showed that improving the level of agricultural technology innovation is conducive to rural economic development. This study provides new insights into the effects of rural financial development on sustainable agricultural development from the perspective of agricultural technology innovation.

## 1. Introduction

The Food and Agriculture Organization of the United Nations (FAO) defines agricultural technology innovation as “the process where by individuals or organizations bring new or existing products, processes, or ways of an organization into use for the first time in a specific context, to increase effectiveness, competitiveness, and resilience to solve a problem.” (Source: The official website of The Food and Agriculture Organization of the United Nations (FAO) http://www.fao.org/innovation/en/). Innovation in agriculture plays a key role in increasing food security and promoting sustainable development [1]. Additionally, food security is the essence of public health. In the report of the 19th National Congress of the Communist Party of China, China proposed a national strategy for a healthy China, namely, “Prevention and Control of Major Diseases: Implementation of a Food Safety Strategy to Ensure People’s Healthy Eating.” (Source: The Official website of the Central People’s Government of the People’s Republic of China. http://www.gov.cn/zhuanti/2017-10/27/content_5234876.htm). In 2017, a series of important policies, such as “Opinions on Promoting Green Agricultural Development through Innovative Institutions and Mechanisms”, were issued to promote innovation in sustainable agricultural development. China has released the “No. 1 Document on Agriculture” for 17 consecutive years since the beginning of the 21st century. The government views the three rural issues of agriculture, the countryside, and farmers as central to economic reconstruction. It focuses more on the development of agricultural science and technology. Therefore, this study explores the impact of rural financial development on agricultural technology innovation in China.

In 2015, the contribution rate of China’s agricultural technology innovation in agricultural production exceeded 56%. The coverage of superior varieties of major crops was stable at 96%, and the total mechanization rate of farming and harvesting reached 65% in 2016 [2]. However, China’s agricultural technology innovation still lags behind industrial nations and shows a significant deficit in innovational input. The data showed that in 2012, the investment intensity in agricultural scientific research in China was 0.77%, while the investment intensity in local governments was even lower at only 0.65% [3], which is much less than the 1% standard set by the FAO. Furthermore, the number of scientific and technological patents owned by agricultural enterprises and the proportion of effective patents to the total number of patent applications is far lower than the average level for all enterprises [4]. Low innovation in agricultural science and technology leads to a series of problems, such as out-of-date technological equipment, low production efficiency, and low value-added agricultural products. This inhibits agricultural science and technology to promote economic development and realize sustainable agricultural development [5]. Simultaneously, insufficient funds for agricultural science and technology research and development can limit agricultural technology innovation capacity [6].

Innovation needs to be supported by a high-quality financial system with more patience and tolerance for failure [7]. Agricultural technology innovation activities also need to be supported. Using panel data from 31 Chinese provinces from 2003 to 2015, this study focused on how rural financial development affects agricultural technology innovation from the perspectives of rural financial scale and rural financial efficiency. It also studied the heterogeneity of the influence of rural financial development on agricultural technology innovation in regions with different levels of marketization and economic development.

Our study makes several contributions to the existing literature. First, it discusses how to promote agricultural technology innovation from finance and technology perspectives, establishing links between agricultural technology innovation and agricultural economic development. Most existing studies focus on the relationship between rural finance and agricultural economic development and conclude that rural finance plays an important role in promoting agricultural economic development. However, few scholars have directly explored the impact of rural financial development on agricultural technology innovation. From the perspective of financial scale and financial efficiency, this study examines the effect of rural financial development on agricultural technology innovation.

Second, this study extends the existing literature on financial and economic development and research on the determinants of agricultural technology innovation. According to the new economic growth theory, knowledge accumulation is the key driving force to promote economic growth and the core element to improve productivity. Economic development cannot be separated from the support of technology innovation and the capital support of the financial system. Scientific and technological innovation is a key link to acquiring new knowledge, producing a knowledge effect, and injecting vitality into economic development. The principal task of the financial sector is to provide services to the real economy and promote its rapid development. This study’s empirical results further show that scientific and technological innovation is also an important mechanism for financial development to promote economic development.

Third, it highlights that the effects of the rural financial scale and efficiency on agricultural technology innovation vary in regions with different marketization and economic development levels, which provide important information for policymakers. The conclusion of this article provides a new way to enhance agricultural technology innovation. Policymakers should promote rural financial development from different regions to stimulate agricultural technology innovation and eventually promote economic development.

## 2. Literature Review, Theoretical Analysis, and Research Hypotheses

### 2.1. Literature Review

#### 2.1.1. Study on the Economic Consequences of Rural Financial Development

As the main driving force of modern rural economic development, rural finance plays a decisive role in agricultural development. The development of rural finance can provide strong financial support for agricultural, scientific, technological research and development, and technological transformation [8]. However, the development of rural finance can effectively improve production and operation [9], directly affecting agricultural production [10]. Most research on rural finance focus on the rural economy [8,11,12], farmers’ income [13,14,15], the poverty reduction effect [16,17,18], and total factor productivity in agriculture [19].

First, although the relationship between finance and the economy is very complex, most scholars believe that rural finance development can promote the development of the agricultural economy [8,20]. In particular, the impact of agricultural policy-based finance on sustainable growth in the agricultural sector varies from region to region [11]. Second, farmers’ income and the relationship between financial development and income distribution [21] were the first to be studied, which showed an inverted U-type relationship. This conclusion was also verified by Banerjee et al. [14] and Ueda et al. [15]. At the same time, financial development can help alleviate inequality in farmers’ income [22], although rural financial development may have a very limited impact on farmers’ income [13,23]. Third, regarding the poverty reduction effect, most scholars agree that rural financial development can play a role in alleviating poverty [6,18], and this promotion effect benefits from the type of economic growth [16]. However, the mitigation effect of finance on poverty may not be absolute. If financial services are only aimed at the rich, they have almost no substantive effect on poverty alleviation [24,25]. Last, rural financial development positively affects the total productivity factor in agriculture, and this effect can be achieved through technological progress [6,15]. Whether financial development can reduce carbon emissions has become a hot research topic in environmental issues. So far, there is no consensus on whether financial development is positively correlated, negatively correlated, or has no significant correlation with carbon emissions [26,27,28].

#### 2.1.2. Research on Determinants of Agricultural Technology Innovation

Owing to the constraints of natural resources and the deterioration of the ecological environment, the development of the modern agricultural economy depends more on the degree of innovation in agricultural science and technology. Therefore, improving the level of agricultural technology innovation has become the main focus of academic research. Scholars mainly focus on three aspects: innovation subjects [29,30], the external environment [31,32,33], and industrial agglomeration [34,35]. First, from the perspective of innovation subjects [25], they must have a certain level of innovation capability to ensure the smooth progress of agricultural technology innovation. Private investment in the scientific research system can also impact agricultural technology innovation [30]. Additionally, the government can also promote scientific research and development by incentivizing agricultural researchers to participate in research and innovation. Second, from the external environment’s perspective, Gilles found that financial exclusion negatively impacts agricultural technology innovation [31]. However, when the mechanism of market adjustment fails, the regulatory role of the government becomes more important and can provide a good external environment for agricultural technology innovation [32]. Additionally, Carletto et al., Havey and Pilgrim, and Patto et al. also examined the impact of climate change, energy deficiency, and economic globalization on agricultural technology innovation [36,37,38], respectively. Finally, from industrial agglomeration, Marshall and Jacobs put forward two competing theories [34,35]. If the Marshall-type externality is dominant, the higher the degree of accumulation of a single industry, the more favorable it is for agricultural innovation development; the driving effect of the individual competitive industry in the region will be the main influencing factor for agricultural technological innovation. If the Jacobs-type externality is dominant, the higher the degree of diversification of industrial structures, the more conducive to agricultural innovation and development; the influencing factors of agricultural technology innovation are mainly environment policies conducive to the diversification of the agricultural industry.

### 2.2. Hypotheses Development

Agricultural technology innovation is risky and costly [39]. Agricultural enterprises and farmers, excluding colleges and research institutions, are important applicants and users of agricultural patents [40]. Agricultural science and technology enterprises need a certain amount of investment in research and development to benefit from agricultural technology innovation. However, owing to the large risks and the long delays in receiving returns on investment, enterprises often experience external financing constraints. They experience great financing pressure if they pursue agricultural technology innovation [41]. As an important part of the national macro-financial system, rural finance can provide financial support to agricultural scientific research institutions, expand educational support, and cultivate numerous professional talents. It can thus play an important role in compensating for the lack of and improving the capacity for agricultural technology innovation [42,43,44]. The development of rural finance can help agricultural enterprises that need funds to access financial support and agricultural enterprises that do not need money to become fund providers. Additionally, digital finance has become a new method of obtaining financial support. Rural finance combined with new digital technology can attract “long tail” customers and provide related services to relax financial restrictions on agricultural science and technology enterprises.

However, for farmers, the adoption of agricultural technology goes through five stages: cognition, persuasion, decision-making, implementation, and confirmation [45]. Rural finance mainly influences farmers’ choice and adoption of new agricultural technologies through the above five stages. It then influences the achievement transformation and the popularization effect of agricultural technology innovation. The development of rural finance can diversify certain risks effectively and reduce the cost of technical tools used by farmers [43,46]. Farmers can take the initiative to understand and accept new agricultural technologies, thus creating more favorable conditions for promoting agricultural technology innovation and transformation. With the gradual implementation of “inclusive finance,” the number of rural financial institutions gradually increases. The development of rural finance can better provide financial services for farmers in financial difficulties and reduce their concerns regarding funds. Additionally, the application of new agricultural technologies can improve production efficiency and support value-added agricultural products. Therefore, farmers may be more inclined to choose new agricultural technologies, thus improving innovation. According to the above analysis, rural financial development can alleviate the external financing constraints of agricultural science and technology enterprises, increase investment in science and technology, and improve the efficiency of capital allocation, but also promote the acceptance and use of new agricultural technologies by farmers, both subjectively and objectively, to improve the promotion and conversion effect of agricultural science and technology achievements. The above discussion leads to our first hypothesis:

**Hypothesis** **1.** *Rural financial development can significantly enhance agricultural technology innovation.*

The rural financial scale and rural financial efficiency are two important aspects of rural financial development. The scale of rural finance is a quantitative concept, which mainly reflects the degree to which the financial system can provide resources for economic operations, thus laying a quantitative foundation for allocating resources in the financial system [47]. Rural financial efficiency is a qualitative concept, which refers to the efficiency and state of resource allocation in the financial system. The improvement of financial efficiency can effectively improve the efficiency of economic operations by finding the most efficient mechanism to allocate resources according to changes in market demand and supply that cause price changes.

In the past decades, China has implemented various market-oriented reforms, which have played a pivotal role in China’s rapid economic development. In the process of marketization, market mechanisms encourage benefit maximization and efficiency optimization, as well as competition and the pursuit of material interests [48]. The degree of marketization is often used to measure how the market contributes to the allocation of resources. The higher the degree of marketization, the higher the efficiency of resource allocation. In regions with a low degree of marketization, a low level of market participation results in low resource allocation efficiency. In the absence of an effective market mechanism, indiscriminately expanding the financial scale and increasing capital input will produce marginal contribution. Therefore, the effective utilization of financial resources and rural financial efficiency improvement are the top priorities to promote agricultural technology innovation in regions with a low degree of marketization. However, in regions with a high degree of marketization, the market mechanism is more developed, and the market plays a greater role in the allocation of resources and other factors. Hence, the efficiency of resource allocation efficiency is relatively high. Additionally, due to the high-efficiency level, the demand for capital and financial services is relatively strong. Therefore, in areas with a high degree of marketization, expanding the scale of rural finance is the primary way to promote agricultural technology innovation by providing sufficient impetus for the demand for capital for agricultural technology innovation and rapid economic development. In summary, our related discussion leads to our second hypothesis:

**Hypothesis** **2.** *The promotion effect of rural financial efficiency on agricultural technology innovation is more significant in regions with a low degree of marketization. The promotion effect of rural financial scale on agricultural technology innovation is more significant in regions with a high degree of marketization.*

## 3. Data Source, Variable Definition, and Research Design

### 3.1. Data Sources and Sample Construction

This study analyzed panel data of 31 provinces, municipalities, and autonomous regions in China from 2003 to 2015. The index data for the rural financial efficiency scale and rural financial scale were collected from the rural financial economy database in the CSMAR database. The data related to innovation were collected manually using the Baiteng patent search system (http://www1.baiten.cn/). The Baiteng patent search system is a free patent search website in China. It includes the patents of 103 countries, regions, and organizations worldwide, with a data volume of 130 million items. The controlled variables in this study were obtained from the CSMAR database and the annual regional data from the official website of the National Bureau of Statistics of China.

### 3.2. Measurement of Variables

Agricultural technology innovation. Following Chemmanur et al. [49] and Tian et al. [50], we use the logarithm of the number of patents as a proxy for innovation. Three types of patents are granted in China: invention, utility model, and design patents [51]. Invention patents are granted for a creative technical solution relating to a product or an improvement. Utility model patents are granted for the shape or structure of a product having new and practical technical solutions. Design patents are granted for the new “look” of a product. This includes new designs relating to the shape, color, pattern, or their combinations, and the new “look” should be aesthetically appealing and industrially applicable. Given the above, we construct our innovation outcome measures using the invention and utility model patents. We measure agricultural technology innovation on three levels: the number of agricultural invention patents, the number of agricultural utility patents, and a total number of two types of patents. Patent refers to the logarithm of the number of agricultural inventions and utility patents plus 1. Invention refers to the logarithm of the number of agricultural invention patents plus 1. Utility refers to the logarithm of the number of agricultural utility patents plus 1.

Rural financial efficiency (RFE). Goldsmith proposed that the Financial Related Ratio (FIR) can be used to measure a country’s financial development level, which is generally accepted by scholars [52]. Rural credit cooperatives are the main source of stable long-term finance in rural areas. They carry out the policy of supporting agriculture with consistency and are less affected by the policy of supporting agriculture ordered by superiors. Therefore, according to Wang [8], we used the deposit-to-loan ratio of rural credit cooperatives in the China Rural Finance Yearbook as a proxy for rural financial efficiency.

Rural Financial Scale (RFS). The Goldsmith index, namely, the ratio between the M2 money supply and gross domestic product (M2/GDP), is used internationally to represent the scale of financial development. Since the main products of rural financial development in China are the deposits and loans of financial institutions, and not financial products such as stocks, bonds, and funds, most Chinese scholars use the ratio between agricultural loans plus township enterprise loans and rural GDP. However, in the China Financial Yearbook after 2008, there are no indicators such as “agricultural loans” and “township and village enterprises loans”. The China Statistical Yearbook 2009 also discontinued the above two indicators and switched to a new statistical category, “Agricultural Loans”. The above two statistical indicators are not comparable and are not continuous. Therefore, referring to Wang, we selected the scale of farmers’ household savings deposits at financial institutions as a proxy for rural financial scale [8], which not only reflects the scale of rural finance but also avoids a strong correlation with the index of the deposit-to-loan ratio of rural credit cooperatives.

### 3.3. Model Specifications

Following Xu and Ruan [53] and Huang et al. [54], to examine the association between rural financial development and agricultural technology innovation, we estimated the following ordinary linear squares regression model for the two measures of rural financial development.

(1) $$\begin{array}{c} \mathrm{Innovation}=\alpha_{0}+\alpha_{1}\mathrm{RF}+\alpha_{2}\mathrm{GFE}+\alpha_{3}\mathrm{Labor}+\alpha_{4}\mathrm{Dep}+\alpha_{5}\mathrm{Dev}+\alpha_{6}\mathrm{FAI}+\alpha_{7}\mathrm{FISA}+\alpha_{8}\mathrm{GDPPerCapita}\\ +\mathrm{Year}+\varepsilon \end{array}$$

where the dependent variable, rural technology innovation, is Patent, Invention, or Utility. The independent variable of rural financial development (RF) is rural financial efficiency (RFE) or rural financial scale (RFS). GFE is financial support for agriculture, expressed as the proportion of agriculture-related expenditure in the fiscal expenditure for each region. Labor refers to the input level of the agricultural labor force, which is measured by the logarithm of the number of employees in agriculture, forestry, animal husbandry, and fisheries. Dep represent the demographic structure, which is defined as the ratio of the population below 15 and above 65 to the population between 15 and 64. Dev is the scale of investment in comprehensive agricultural development projects of each province; FAI is the logarithm value of the total social investment of each province. FISA represents government participation in the economy, which is reflected in local government spending as a percentage of GDP and plays an important role in developing China’s agricultural economy. GDPPerCapita represents the economy development of the provinces, which is per capita GDP of provinces. Table 1 provides detailed variable definitions. At the same time, the time fixed effects are controlled to control for variables that are constant across entities but vary over time.

## 4. Empirical Results

### 4.1. Descriptive Statistics

Table 2 summarizes the descriptive statistics for the variables. It shows that the average number for Utility was 4.853. The number of agricultural invention patents was 3.912, which means that the number of invention patents was less than the number of utility patents in China’s agricultural development. The mean value of the Rural Financial Efficiency (RFE) Index was 1.481, the highest value was 2.254, and the minimum value was 1.048. The mean value of the Rural Financial Scale (RFS) Index was 1.537, the highest value was 9.476, and the minimum value was 0.246. The GFE is the strength of agricultural financial support. The average value was 0.079, the highest value was 0.171, and the minimum value was 0.079, which also reflects that China’s agricultural financial support needs to be further strengthened to some extent.

### 4.2. Analysis of the Main Results

A summary of the results is shown in Table 3. The RFE was positively correlated with Patent and Invention at a significance level of 1%, and the coefficients were 0.545 and 0.606, respectively. Utility had a positive correlation at the 5% significance level and a coefficient of 0.420. RFS was positively correlated with Patent, Invention, and Utility with a 1% significance level and coefficients of 0.340, 0.428, and 0.293, respectively. The above data were based on the two dimensions of rural financial efficiency and rural financial scale, which verified Hypothesis 1, namely, that rural financial development could significantly enhance the strength of agricultural technology innovation. The main reasons are that the improvement in rural financial efficiency is conducive to the effective allocation of financial resources, improves the promotion and conversion effect of new agricultural technologies and that the expansion of rural financial scale alleviates the external financing constraints of enterprises and increases investment in science and technology, which is conducive to the development of enterprises’ scientific research activities.

The rural financial efficiency and the scale of rural finance are affected by the degree of marketization. The promotion effect on the development of agricultural science and technology also shows different characteristics. We use the National Economic Research Institute of China Reform Foundation (NBRI) index of marketization to measure the marketization process of regions in China. This index measures marketization progress from five aspects, namely, the relationship between government and market, development of the non-state (private) sector, development of product markets, development of factor markets, and the development of market intermediaries as well as the market-friendly legal environment. This system is constructed based on objective statistics or survey data [55]. This index can provide rich information on the process of marketization and has been widely used in the existing literature [56,57,58]. The marketization index data from 2008 to 2015 were selected for analysis. We assumed that the degree of marketization did not change during 2003–2008 and the value of the marketization index before 2008 is replaced by the value of 2008. We grouped the sample with a low degree of marketization if the marketization index of the region is lower than the sample median and high degree of marketization otherwise. Therefore, this study divided the sample into high and low degrees of marketization for the group test and investigated the differential influence of rural financial efficiency and rural financial scale on agricultural technology innovation.

We also do some robustness tests. We adopted one-period and two-period lagged explanatory variables for regression considering the long cycle of scientific and technological innovation and the lag effect of rural financial development on agricultural technology innovation. There is a significant positive correlation between one-period lagged RFE and RFS with agricultural technology innovation (Patent, Invention, and Utility). The regression results are stable. This indicates that the research conclusion of this study is highly robust. Table 4 summarizes the impact of financial efficiency on agricultural technology innovation in different degrees of marketization. There was no significant positive correlation between RFE and the innovation indicators (Patent, Invention, and Utility) in the groups with a high degree of marketization. However, in the groups with a low degree of marketization, RFE was positively correlated with innovation (Patent, Invention, and Utility) at a significance level of 1%, with coefficients of 1.284, 1.286, and 1.186, respectively. This indicates that the promotion effect of rural financial efficiency on agricultural technology innovation was more significant in areas with a low degree of marketization, which supported Hypothesis 2. In regions with a low degree of marketization, resource allocation efficiency was not high due to a lack of a relatively complete market mechanism leading to unreasonable allocation and inadequate utilization of many resources. Therefore, in this case, improving rural financial efficiency, allocating resources reasonably according to market supply, and giving full play to the maximum benefit of existing resources would greatly promote agricultural technology innovation in this region.

Table 5 summarizes the impact of financial scale on agricultural technology innovation under different degrees of marketization. The positive correlation between RFE and the innovation indicators (Patent, Invention, and Utility) was not significant in groups with a low degree of marketization. However, in the groups with a high degree of marketization, there was a positive correlation between RFE and the innovation indicators (Patent, Invention, and Utility) at a significance level of 1%, coefficients of 0.474, 0.546, and 0.416, respectively. This indicates that the promotion effect of the rural financial scale on agricultural technology innovation was more significant in regions with a high degree of marketization, verifying Hypothesis 2. This is because regions with a higher degree of marketization have a higher capacity, resource allocation, and operation level. They can effectively and rationally invest resources in high demand areas, thus contributing to the economy’s efficient operation. Therefore, with a strong resource allocation ability, expanding the scale of rural finance, actively meeting the capital demand of agricultural technology innovation, and increasing investment in agricultural science and technology research and development can effectively promote agricultural technology innovation levels.

## 5. Further Discussion

### 5.1. Agricultural Technology Innovation, Agricultural Economic Development

Agricultural technology innovation is closely related to agricultural economic development. The food and agriculture sector is expected to provide healthy, safe, and nutritious food for a growing population. However, agriculture is resource-intensive, accounting for about a quarter of global greenhouse gas emissions and 70% of the global demand for fresh-water. The capacity of agricultural innovation systems is key to meeting these challenges, improving farm productivity and environmental performance, and contributing to public health. It is worth contemplating whether innovation in agricultural science and technology can promote the development of the agricultural economy and form a virtuous circle to better serve the sustainable development of rural areas. Therefore, this study explored the relationship between agricultural technology innovation and the development of the agricultural economy.

Table 6 summarizes the results, where Lngap is the logarithm of the total value of farm output. Patent was positively correlated with Lngap at a significance level of 1% and a coefficient of 0.210. Invention and Lngap were positively correlated at a significance level of 5%, with a coefficient of 0.070. Utility was positively correlated with Lngap at a significance level of 1%, with a coefficient of 0.216. This shows that agricultural technology innovation can promote agricultural economic development. The result suggests that agricultural technology innovation can ease concerns about resource scarcity and the possible starvation of an expanding world population. Furthermore, agricultural technology innovation can also improve the efficiency of resources and reduce ecological impact and a smaller carbon footprint, improving the quality of our lives.

### 5.2. Regional Heterogeneity

The developmental imbalance between the eastern and western regions is a unique situation in China. Rural financial development also has a different impact on the innovation of agricultural science and technology due to this variation in development levels. Therefore, based on the context of China, this study divided the whole sample into eastern and western regions. Based on the two perspectives of rural financial efficiency and rural financial scale, this paper discusses the impact of rural financial efficiency on agricultural technology innovation in these two groups. The eastern region included Beijing, Tianjin, Hebei, Shanghai, Jiangsu, Zhejiang, Fujian, Shandong, Guangdong, and Hainan. In contrast, the western region included Chongqing, Sichuan, Guizhou, Yunnan, Shaanxi, Gansu, Qinghai, Ningxia, Xinjiang, and Tibet.

Table 7 shows that in the eastern region, the positive correlation between RFE and innovation indicators (Patent, Invention, and Utility) is not significant. However, in the western region, there is a positive correlation between RFE and Patent and Utility at a significance level of 1%, with coefficients of 1.093 and 1.240, respectively; however, there is no significant positive correlation between RFE and Invention. Compared with the eastern region, rural financial efficiency plays a more significant role in promoting agricultural technology innovation in the western region. This is because the western region’s economic development level is relatively backward, and marketization is low. Hence, the ability and efficiency of resource allocation are also low. Therefore, improving rural financial efficiency, focusing on the reasonable allocation of existing resources, and further optimizing the resource allocation will significantly promote agricultural technology innovation in this region.

Table 8 shows that RFS and Patent are positively correlated in the eastern region at a 1% significance level, with a coefficient of 0.384; and RFS and Invention and Utility are positively correlated at 5% significance level, with coefficients of 0.349 and 0.385, respectively. However, the negative correlation between RFS and innovation indicators (Patent, Invention, and Utility) is not significant in the western region. Compared with the western region, the scale of rural finance plays a more significant role in promoting agricultural technology innovation in the eastern region. This can generally be attributed to the eastern region’s high development level, favorable conditions for resource allocation, and economic development. The effective market operation and relatively developed market environment also create a high demand for capital. Therefore, expanding the scale of rural finance and actively meeting the capital requirements of agricultural development in the eastern region has a significant positive effect on innovation in agricultural science and technology in the eastern region.

## 6. Conclusions

In this study, we discuss how to promote agricultural technology innovation in China from a finance and technology perspectives, whilst establishing links between agricultural technology innovation and agricultural economic development. Based on the panel data of 31 provinces in China from 2003 to 2015, we find that rural finance development has a significant positive effect on agricultural technology innovation. The effect of rural financial efficiency on agricultural technology innovation is more significant in regions with a low degree of marketization as evident in the western region. In contrast, the promotion effect of the rural financial scale on agricultural technology innovation is more significant in regions with a high degree of marketization, such as the eastern region. It was also revealed that agricultural technology innovation is conducive to agricultural economic development and can better promote sustainable rural development. This study extends the existing literature on financial and economic development and research on the determinants of agricultural technology innovation. It highlights that the effects of the rural financial scale and efficiency on agricultural technology innovation vary in regions with different marketization and economic development levels, which provide important information for policymakers, especially on how to promote agricultural technology innovation.

This study has important implications for both theory and practice. On a theoretical level, unlike previous studies that mainly focused on exploring rural finance development and the relationship between agricultural economic development, based on the internal logic of scientific and technological innovation’s promotion of economic development, this study sets up a logical relationship between the level of agricultural technology innovation and rural financial development. This complements the determinants of agricultural technology innovation and further enriches the existing literature on financial development and economic development.

This study’s research conclusions have important implications for both enterprises and government policymakers at the practical level. In recent years, the Chinese government has emphasized the “Agriculture, Countryside, and Farmers,” placed them first in economic construction, and focused on agricultural science and technology development. The Chinese government has implemented many policies to encourage rural financial institutions to provide financial support for agricultural development-related programs, especially those related to agricultural technology innovation. From the perspective of enterprises, this research can, to a certain extent, encourage relevant agricultural enterprises to make full use of government policies to obtain financial support from rural financial institutions and achieve innovation and improve their competitiveness in the future.

This study’s empirical results show that the rural financial scale plays a more significant role in promoting agricultural technology innovation in developed regions. In contrast, in less developed regions, rural financial efficiency plays a more significant role in promoting agricultural technology innovation. Therefore, when formulating relevant policies, the government should make decisions based on local conditions, and improve and perfect the rural financial system based on the different characteristics of different regions, to promote agricultural technology innovation better. However, agricultural technology innovation is key to balancing agricultural productivity and environmental performance in the agricultural sector to improve public health. Therefore, the government should exploit the different kinds of green financial incentive mechanisms, explore effective methods to develop green agriculture with green financial services, increase green credit support and specialized guarantees, and make innovations in green and ecological agricultural insurance products. Cooperation between the government and public-private partnerships (PPP) can also be promoted and applied in agricultural development, guiding the social investments to agricultural resource conservation, waste resource reuse, animal epidemic prevention, and ecosystem restoration, thus providing credit for high-quality agricultural technology innovation.

This study raises important avenues for further study. We examined the relationship between the rural financial development and agricultural technology innovation. Rural financial system needs innovation as it is important to rural revitalization. Therefore, how to design and promote the rural financial system in the process of rural economic development is an important topic. Considering a series of natural, market, and institutional factors, agricultural business entities always find it difficult to obtain credit from most financial institutions largely due to the mismatch between risks and returns. For example, agricultural production is severely restricted by uncontrollable climate change, natural disasters, and other factors. The output volatility is large, and the profitability is difficult to accurately predict, which is not conducive to financial institutions to provide financing services for agricultural production. However, the fund of rural financial institutions is vital to rural technological innovation and rural economic development. Furthermore, the diversification of agricultural and rural economic entities determines that rural financial system must be diversified and multi-layered. How to carry out rural financial system innovation, and then promote the financial services in rural technological innovation and rural economic development, is an important proposition for future research. In addition, many policy financial instruments must be used in the innovation of future rural financial system, and this study only touches on the impact of rural financial development on rural technological innovation. Therefore, the impact of various financial instruments on rural technological innovation and rural economic development represents an important topic for future research.

## Figures and Tables

**Table 1:** Variables Definition.

Variable Name	Variable Definitions
Patent	Log (1+ number of agricultural invention and utility model patents)
Invention	Log (1+ number of agricultural invention patents)
Utility	Log (1+ number of agricultural utility model patents)
RFE	Rural financial efficiency is referred to Wang Jinyi, by the credit ratio of rural credit cooperatives [8].
RFS	The scale of rural finance is expressed by the natural logarithm of farmers households’ savings deposits Wang Jinyi for reference [8].
GFE	The intensity of financial support for agriculture is expressed by the proportion of agriculture-related expenditures in the fiscal expenditure of each region.
Labor	The input level of agricultural labor force is measured by the logarithm of the number of practitioners in agriculture, forestry, animal husbandry and fishery.
Dep	Demographic structure is defined as the ratio of the population below 15 and above 65 to the population between 15 and 64.
Dev	The scale of investment in comprehensive agricultural development projects of each province shall be expressed by the natural logarithm of the total amount of investment in comprehensive agricultural development projects of each province, including funds from the central finance, supporting funds from local finance, bank loans and self-raised funds.
FAI	The total fixed investment of the whole society, the logarithm value of the total social investment of each province.
FISA	Represents the participation of the government in economic activities. Local governments in China play an important role in the development of agricultural economy. The proportion of the total fiscal expenditure of local governments in the gross domestic product reflects the participation of local governments in economic activities.
GDPPerCapita	The logarithm of Per capita GDP of provinces

**Table 2:** Descriptive Statistics (The sample size in descriptive statistics is the sample participating in the main regression).

Variable	*N*	Mean	SD	Min	p25	p50	p75	Max
Patent	297	5.211	1.177	2.197	4.454	5.247	5.999	7.989
Invention	297	3.911	1.292	0.693	2.996	3.989	4.820	6.850
Utility	297	4.853	1.168	1.792	4.094	4.868	5.638	7.603
RFE	279	1.479	0.180	1.017	1.369	1.470	1.586	1.955
RFS	287	6.736	1.145	3.240	6.065	6.803	7.545	8.958
GFE	297	0.079	0.042	0.016	0.038	0.082	0.117	0.169
Labor	297	7	1.015	4.347	6.335	7.242	7.787	8.489
Dep	297	0.376	0.065	0.246	0.334	0.378	0.420	0.547
Dev	297	11.57	0.646	10.08	11.12	11.62	12.08	12.82
FAI	297	8.269	1.053	5.667	7.540	8.328	9.077	10.51
FISA	297	0.194	0.092	0.0840	0.132	0.169	0.229	0.585
GDPPerCapital	297	9.904	0.643	8.190	9.433	9.914	10.39	11.28

**Table 3:** Rural Financial Development and Agricultural Technology Innovation.

	(1)	(2)	(3)	(4)	(5)	(6)
	Patent	Invention	Utility	Patent	Invention	Utility
RFE	0.545 ***	0.606 ***	0.420 **			
	(3.617)	(3.222)	(2.389)			
RFS				0.340 ***	0.428 ***	0.293 ***
				(3.904)	(3.403)	(3.601)
GFE	4.387 **	5.560 **	3.466 *	1.073	1.719	0.539
	(2.418)	(2.340)	(1.700)	(0.547)	(0.639)	(0.271)
Labor	0.306 ***	0.433 ***	0.282 ***	0.146	0.243 *	0.134
	(3.153)	(3.393)	(2.807)	(1.434)	(1.958)	(1.286)
Dep	3.961 ***	3.148 ***	4.037 ***	3.754 ***	3.210 ***	3.679 ***
	(5.364)	(3.718)	(4.970)	(5.178)	(4.004)	(4.579)
Dev	0.155	0.523 ***	0.029	0.047	0.401 ***	0.120
	(1.523)	(3.969)	(0.273)	(0.473)	(3.333)	(1.170)
FAI	0.596 ***	0.622 ***	0.535 ***	0.293 *	0.237	0.290 *
	(4.304)	(3.258)	(3.752)	(1.929)	(1.111)	(1.959)
FISA	1.172 *	1.003	1.212 *	0.778	0.311	0.942
	(1.795)	(1.140)	(1.884)	(1.087)	(0.328)	(1.341)
GDPPerCapita	0.260	0.498 **	0.217	0.321 **	0.594 ***	0.245
	(1.581)	(2.299)	(1.266)	(2.117)	(2.855)	(1.597)
Year Fixed Effects	Yes	Yes	Yes	Yes	Yes	Yes
constant	1.900	2.863	3.045	1.616	2.606	2.730
	(0.916)	(1.070)	(1.406)	(0.800)	(0.999)	(1.311)
*N*	279	279	279	287	287	287
r2_a	0.869	0.780	0.856	0.852	0.771	0.842
F	102.612	60.484	88.684	86.450	61.241	75.825

Note: t statistics in parentheses * *p* < 0.1, ** *p* < 0.05, *** *p* < 0.01.

**Table 4:** Financial Efficiency, Marketization Degree, and Agricultural Technology Innovation.

	(1)	(2)	(3)	(4)	(5)	(6)
	High Marketization Degree			Low Marketization Degree		
RFE	0.304	0.354	0.272	1.284 ***	1.286 ***	1.186 ***
	(1.098)	(1.001)	(1.011)	(6.652)	(5.221)	(5.087)
GFE	3.868	2.216	4.428 *	6.576 **	3.113	7.139 **
	(1.616)	(0.659)	(1.815)	(2.340)	(0.836)	(2.166)
Labor	0.338 **	0.311 **	0.370 ***	0.063	0.458 **	0.271
	(2.502)	(2.021)	(2.663)	(0.396)	(2.399)	(1.505)
Dep	2.904 ***	1.603	3.263 ***	8.219 ***	7.092 ***	8.502 ***
	(3.226)	(1.413)	(3.190)	(7.629)	(4.943)	(7.889)
Dev	0.142	0.004	0.237 *	0.317 *	0.754 **	0.077
	(1.245)	(0.038)	(1.905)	(1.737)	(2.496)	(0.391)
FAI	0.632 ***	0.717 ***	0.557 ***	0.728 ***	0.326	0.830 ***
	(3.314)	(3.413)	(2.868)	(3.939)	(1.213)	(3.942)
FISA	1.797	4.000 **	0.866	3.369 ***	4.150 ***	3.177 ***
	(1.367)	(2.371)	(0.660)	(4.773)	(4.308)	(4.400)
GDPPerCapita	0.655 **	1.183 ***	0.487 *	1.541 ***	1.198 ***	1.739 ***
	(2.570)	(4.070)	(1.856)	(4.840)	(2.872)	(4.835)
Year Fixed Effects	Yes	Yes	Yes	Yes	Yes	Yes
constant	9.610 ***	15.632 ***	8.809 ***	18.525 ***	17.892 ***	18.238 ***
	(3.431)	(5.278)	(2.899)	(5.544)	(3.725)	(5.044)
*N*	133	133	133	146	146	146
r2_a	0.887	0.854	0.871	0.890	0.794	0.874
F	62.046	54.454	50.976	81.489	38.830	73.282

Note: t statistics in parentheses * *p* < 0.1, ** *p* < 0.05, *** *p* < 0.01.

**Table 5:** Financial Scale, Degree of Marketization, and Agricultural Technology Innovation.

	(1)	(2)	(3)	(4)	(5)	(6)
	High Marketization Degree			Low Marketization Degree		
RFS	0.474 ***	0.546 ***	0.416 ***	0.131	0.022	0.185
	(4.745)	(4.018)	(4.242)	(1.076)	(0.138)	(1.550)
GFE	3.212	0.809	4.085	8.319 **	5.348	8.530 **
	(1.330)	(0.266)	(1.611)	(2.595)	(1.134)	(2.523)
Labor	0.220 *	0.162	0.259 **	0.063	0.621 **	0.178
	(1.921)	(1.128)	(2.328)	(0.290)	(2.600)	(0.765)
Dep	2.828 ***	1.704	3.111 ***	6.996 ***	6.042 ***	7.255 ***
	(3.527)	(1.571)	(3.485)	(5.439)	(4.019)	(5.549)
Dev	0.236 **	0.126	0.298 ***	0.068	0.491	0.153
	(2.539)	(1.207)	(2.976)	(0.261)	(1.411)	(0.558)
FAI	0.113	0.106	0.140	0.457 *	0.140	0.531 *
	(0.732)	(0.514)	(0.958)	(1.743)	(0.395)	(1.900)
FISA	3.697 ***	5.613 ***	2.722 ***	2.741 ***	3.533 ***	2.553 ***
	(4.113)	(5.128)	(2.979)	(3.604)	(3.549)	(3.186)
GDPPerCapita	0.831 ***	1.353 ***	0.621 ***	0.859 ***	0.557	1.083 ***
	(4.564)	(5.931)	(3.375)	(2.625)	(1.250)	(3.146)
Year Fixed Effects	Yes	Yes	Yes	Yes	Yes	Yes
constant	10.433 ***	16.088 ***	9.377 ***	11.139 ***	10.536 **	11.282 ***
	(4.463)	(6.105)	(3.726)	(3.269)	(2.134)	(3.195)
*N*	147	147	147	140	140	140
r2_a	0.886	0.858	0.868	0.831	0.740	0.815
F	99.578	67.176	51.425	52.553	32.222	53.282

Note: t statistics in parentheses * *p* < 0.1, ** *p* < 0.05, *** *p* < 0.01.

**Table 6:** Technology Innovation and Agricultural Economic Development.

	(1)	(2)	(3)
	Lngap	Lngap	Lngap
Patent	0.210 ***		
	(4.646)		
Invention		0.070 **	
		(2.384)	
Utility			0.216 ***
			(5.011)
GFE	4.999 ***	5.123 ***	5.085 ***
	(3.580)	(3.761)	(3.599)
Labor	0.271 ***	0.303 ***	0.275 ***
	(4.020)	(4.294)	(4.097)
Dep	0.749	0.141	0.770
	(1.539)	(0.283)	(1.606)
Dev	0.429 ***	0.437 ***	0.393 ***
	(6.456)	(6.281)	(5.994)
FAI	0.147	0.225 **	0.151
	(1.450)	(2.244)	(1.559)
FISA	3.011 ***	3.179 ***	2.993 ***
	(6.592)	(7.471)	(6.539)
GDPPerCapita	0.330 **	0.303 **	0.316 **
	(2.530)	(2.378)	(2.412)
YearFixedEffects	Yes	Yes	Yes
constant	0.956	0.807	1.203
	(0.610)	(0.531)	(0.769)
*N*	297	297	297
r2_a	0.915	0.908	0.916
F	179.489	166.282	181.781

Note: t statistics in parentheses ** *p* < 0.05, *** *p* < 0.01.

**Table 7:** Financial Efficiency, Regional Distribution, and Agricultural Scientific and Technological Innovation.

	(1)	(2)	(3)	(4)	(5)	(6)
	The Eastern Region			The Western Region		
RFE	0.394	0.309	0.398	1.093 ***	0.426	1.240 ***
	(1.191)	(0.837)	(1.186)	(5.363)	(1.298)	(4.568)
GFE	6.743	6.085	6.153	6.534 **	11.428 **	3.332
	(1.486)	(1.431)	(1.142)	(2.342)	(2.537)	(1.002)
Labor	0.384	0.358	0.416	0.376 **	0.012	0.607 ***
	(1.468)	(1.282)	(1.558)	(2.131)	(0.039)	(2.989)
Dep	0.386	0.820	0.372	1.884 *	5.388 **	0.202
	(0.252)	(0.480)	(0.234)	(1.828)	(2.630)	(0.149)
Dev	0.065	0.026	0.047	0.311	0.019	0.497
	(0.287)	(0.129)	(0.187)	(1.132)	(0.052)	(1.550)
FAI	0.480 *	0.446	0.451	0.990 ***	1.294 **	0.866 ***
	(1.800)	(1.610)	(1.637)	(3.874)	(2.594)	(2.666)
FISA	0.983	5.208 **	1.176	3.953 ***	0.128	5.804 ***
	(0.562)	(2.547)	(0.586)	(3.701)	(0.086)	(4.359)
GDPPerCapita	1.159 ***	1.817 ***	0.928 **	1.285 ***	1.027	1.578 ***
	(2.812)	(3.850)	(2.266)	(3.668)	(1.514)	(3.897)
YearFixedEffects	Yes	Yes	Yes	Yes	Yes	Yes
constant	12.817 ***	21.163 ***	10.939 **	7.502 **	3.997	9.611 ***
	(2.893)	(4.060)	(2.449)	(2.331)	(0.616)	(2.663)
*N*	87	87	87	85	85	85
r2_a	0.887	0.856	0.879	0.940	0.862	0.900
F	39.337	22.544	31.215	103.231	45.126	42.307

Note: t statistics in parentheses * *p* < 0.1, ** *p* < 0.05, *** *p* < 0.01.

**Table 8:** Financial Scale, Regional Distribution, and Agricultural Technology Innovation.

	(1)	(2)	(3)	(4)	(5)	(6)
	The Eastern Region			The Western Region		
RFS	0.384 ***	0.349 **	0.385 **	0.070	0.184	0.070
	(2.661)	(2.143)	(2.510)	(0.309)	(0.636)	(0.242)
GFE	3.866	5.619	2.664	7.918 **	12.161 **	4.688
	(0.981)	(1.571)	(0.565)	(2.487)	(2.624)	(1.261)
Labor	0.172	0.159	0.194	0.633 ***	0.035	0.897 ***
	(0.686)	(0.570)	(0.776)	(2.760)	(0.143)	(3.283)
Dep	0.083	0.709	0.163	1.355	4.872 ***	0.375
	(0.060)	(0.456)	(0.115)	(1.275)	(2.884)	(0.261)
Dev	0.151	0.174	0.134	0.269	0.052	0.465
	(0.889)	(0.930)	(0.764)	(0.902)	(0.141)	(1.354)
FAI	0.133	0.096	0.152	1.716 ***	1.716 ***	1.664 ***
	(0.588)	(0.365)	(0.656)	(4.558)	(3.170)	(3.589)
FISA	2.330	5.111 ***	0.541	2.434 **	0.026	4.058 ***
	(1.217)	(2.764)	(0.252)	(2.414)	(0.018)	(3.686)
GDPPerCapita	1.192 ***	1.803 ***	0.946 ***	1.414 ***	1.031	1.724 ***
	(3.673)	(4.697)	(3.048)	(3.372)	(1.476)	(3.964)
YearFixedEffects	Yes	Yes	Yes	Yes	Yes	Yes
constant	13.475 ***	20.918 ***	11.485 ***	7.137 *	3.558	9.146 **
	(3.960)	(4.970)	(3.591)	(1.830)	(0.553)	(2.256)
*N*	95	95	95	88	88	88
r2_a	0.867	0.841	0.860	0.926	0.875	0.884
F	34.329	23.785	27.107	141.652	49.421	74.226

Note: t statistics in parentheses * *p* < 0.1, ** *p* < 0.05, *** *p* < 0.01.

## Data Availability

Not applicable.
